# Recent Trends and Innovations in Bead-Based Biosensors for Cancer Detection

**DOI:** 10.3390/s24092904

**Published:** 2024-05-01

**Authors:** Hui-Pin Cheng, Tai-Hua Yang, Jhih-Cheng Wang, Han-Sheng Chuang

**Affiliations:** 1 Department of Biomedical Engineering, National Cheng Kung University, Tainan 701, Taiwanyangtaihua@mail.ncku.edu.tw (T.-H.Y.); 2 Department of Orthopedic Surgery, National Cheng Kung University Hospital, Tainan 704, Taiwan; 3 Medical Device Innovation Center, National Cheng Kung University, Tainan 701, Taiwan; 4 Department of Urology, Chimei Medical Center, Tainan 710, Taiwan; 5 Department of Electrical Engineering, Southern Taiwan University of Science and Technology, Tainan 710, Taiwan; 6 School of Medicine, College of Medicine, National Sun Yat-sen University, Kaohsiung 804, Taiwan

**Keywords:** bead-based biosensors, cancer, biomarker, electrochemical bead, magnetic beads, optical beads

## Abstract

Demand is strong for sensitive, reliable, and cost-effective diagnostic tools for cancer detection. Accordingly, bead-based biosensors have emerged in recent years as promising diagnostic platforms based on wide-ranging cancer biomarkers owing to the versatility, high sensitivity, and flexibility to perform the multiplexing of beads. This comprehensive review highlights recent trends and innovations in the development of bead-based biosensors for cancer-biomarker detection. We introduce various types of bead-based biosensors such as optical, electrochemical, and magnetic biosensors, along with their respective advantages and limitations. Moreover, the review summarizes the latest advancements, including fabrication techniques, signal-amplification strategies, and integration with microfluidics and nanotechnology. Additionally, the challenges and future perspectives in the field of bead-based biosensors for cancer-biomarker detection are discussed. Understanding these innovations in bead-based biosensors can greatly contribute to improvements in cancer diagnostics, thereby facilitating early detection and personalized treatments.

## 1. Introduction

### 1.1. Cancer-Cell Biology

Cancer is a complex and heterogeneous disease that requires early detection and accurate diagnosis for effective treatment and improved patient outcomes [1]. The complex biology of cancer cells is fundamental to comprehending cancer and developing effective detection and treatment methods. Cancer is characterized by the abnormal and uncontrolled growth of cells, as opposed to normal cells with a regulated lifecycle. The evasion of regulatory mechanisms by cancer cells leads to tumor formation. To thoroughly understand cancer cell biology, several crucial aspects need to be considered. First, genetic mutations play a vital role in cancer development. Accumulated mutations in a cell can disrupt normal regulatory mechanisms, resulting in uncontrolled cell division [2]. Thus, identifying specific genetic alterations in cancer cells has become a fundamental aspect of cancer research. Second, a critical aspect of cancer-cell biology is metastasis. One of the most fatal characteristics of cancer is its ability to spread to other parts of the body. This process is called metastasis. It involves cancer cells breaking away from the primary tumor, entering the bloodstream, and establishing secondary tumors in distant sites [3]. Understanding the molecular and cellular mechanisms driving metastasis is pivotal in fighting against cancer. Cancer is not a single disease, but rather a collection of disorders with unique characteristics. This heterogeneity poses significant challenges in cancer treatment. Intra-tumor heterogeneity, which refers to diverse cell populations within a single tumor, leads to distinct behaviors and responses to therapy among different populations [4]. Understanding the intricate biology of cancer cells is crucial to comprehending cancer and developing effective detection and treatment methods. Genetic mutations, metastasis, and intra-tumor heterogeneity are key aspects to consider in this complex field of research. By unraveling the mechanisms underlying these phenomena, we can improve outcomes for cancer patients.

### 1.2. Cancer Biomarkers

**Figure 1:**
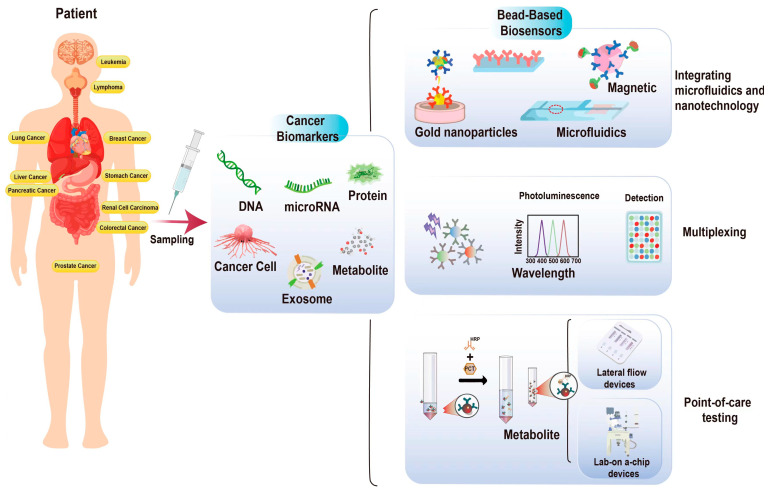
Schematic of circulating cancer biomarkers frequently identified in liquid biopsy, showcasing examples of contemporary trends and innovations in device technology.

Cancer biomarkers are molecules or substances that indicate cancer’s presence, growth, or spread. They can be found in various bodily fluids, such as blood, urine, or tissue samples. These biomarkers can be molecular entities such as proteins, nucleic acids, metabolites, or tumor cells that are specific to cancer or exhibit altered expression levels under cancerous conditions [5,6]. Detecting and analyzing these biomarkers provides valuable insights into the presence, progression, and response to therapy in various types of cancer [7] (Figure 1).

Owing to statistical limitations and challenges related to biochemical factors, using a single biomarker as a predictive or diagnostic marker for cancer is challenging particularly when lacking specificity for a specific disease. Current research indicates the presence of numerous biomarkers associated with various types of cancer. In clinical practice, a combination of biomarkers and clinical indications is necessary to assess disease progression accurately. Consequently, utilizing a single biomarker for disease diagnosis is difficult. For example, prostate-specific antigen (PSA) levels can elevate in prostate cancer and also under benign prostate conditions, leading to diagnostic uncertainties. Consequently, the influential early detection of cancers relies on the simultaneous evaluation of groups of biomarkers. Assessing the levels of four to ten biomarkers is likely to provide more statistically robust prognostic information and increased diagnostic value.

In the clinical setting, cancer patients may concurrently exhibit alterations in various biomarkers, such as changes in inflammatory factors and PIK3CA gene mutations in peripheral blood ctDNA of breast cancer patients. In this context, developing methodologies that enable the simultaneous measurement of these biomarkers in a single detection assay is essential. Multiplexed assays should be able to detect an analyte within the cancer panel with a significantly distinct concentration from the others while maintaining the accuracy of detecting the remaining biomarkers, particularly the crucial cut-off values for distinguishing between cancer and healthy subjects. Currently, some common protein biomarkers are associated with different types of cancers (Table 1).

Cancer biomarkers are indispensable tools in oncology because they play a vital role in diagnosing, monitoring, and predicting the disease. These biomarkers furnish valuable information that can guide treatment decisions and enhancing patient outcomes. The significance of the cancer biomarkers is highlighted as follows. First, diagnostic biomarkers have been proven to be valuable in early cancer detection. These biomarkers can indicate the presence of cancer cells or specific genetic alterations associated with cancer. For instance, PSA in the blood is a diagnostic biomarker for prostate cancer. Detecting PSA in the blood raises suspicion of prostate cancer, prompting the need for further diagnostic tests to confirm the diagnosis. Second, some prognostic biomarkers can provide information about the likely trajectory of the disease. These biomarkers can help determine the aggression of the cancer and the likelihood of recurrence. To illustrate, we take HER2 protein as an example. It has been identified as a prognostic biomarker for breast cancer. Elevated levels of HER2 indicate a more significant potential for more aggressive disease, enabling better treatment decisions and targeted interventions. Finally, predictive biomarkers are particularly important in guiding treatment decisions by indicating how a patient is likely to respond to specific therapies. These biomarkers help personalize treatment plans, ensuring patients receive the most effective interventions with minimal side effects. For example, epidermal growth factor receptor (EGFR) mutations have been identified as predictive biomarkers for the response to targeted therapies in lung cancer. Patients with EGFR mutations are more likely to benefit from these therapies, making them ideal candidates for targeted treatment approaches.

Despite the significant advancements in cancer research, detecting cancer biomarkers still presents numerous challenges. Traditional diagnostic methods including immunoassays and molecular techniques often suffer from the limitations of low sensitivity and limited multiplexing capabilities, as well as the requirement for sophisticated operations [27,28]. These methods are also often time consuming, expensive, and labor intensive. Thus, they are less suitable for routine clinical applications and point-of-care (POC) settings [29,30].

### 1.3. Methodology

For the selection of articles included in this review, a search concerning bead-based biosensors was implemented by looking up keywords, titles, and abstracts of papers. Articles were selected if their abstracts indicated a relationship between cancer biomarkers, bead-based biosensors, and nanoparticles. Only original research articles, review papers, and other scholarly publications written in English were considered for inclusion in this review. The literature search was performed across multiple scientific databases, including PubMed, Scopus, IEEE Xplore, ScienceDirect, and Google Scholar. We used the following search terms: “biomarker” OR “Cancer biomarker” OR “Biosensor” OR “nano-biosensor” OR “Bead-Based immunoassay” OR “immunoassay” OR “Bead” OR “particle” OR “nanoparticle” OR “nanoparticles” in combination with a Boolean operator “OR” to carry out the task.

## 2. Bead-Based Biosensors

### 2.1. Significance of Bead-Based Biosensors

In the ever-evolving landscape of cancer diagnostics, bead-based detection technologies have emerged as a promising frontier. They offer a spectrum of advantages that have redefined possibilities in biomarker detection. At the heart of their efficacy is a high surface-to-volume ratio, rapid response, and high throughput, which have propelled these technologies to the forefront of cancer research. The ability to achieve simultaneous multiplexed detection, process large sample volumes with automation, and maintain high sensitivity and specificity positions bead-based biosensors as indispensable tools in both research and clinical settings. However, these advantages come with accompanying challenges, such as the standardization of protocols, validation of clinical utility, and reproducibility across different settings, all of which require careful consideration. See Table 2. The three principal advantages of bead-based detection technology are discussed below.

#### 2.1.1. High Surface-to-Volume Ratio

Bead-based biosensors possess a significant attribute that contributes to their effectiveness, that is, a high surface-to-volume ratio. This feature plays a pivotal role in enhancing sensitivity and efficiency in detecting cancer biomarkers. The increased immobilization of capture molecules on the bead’s surface, enabled by this feature, allows for a higher density of immobilized capture molecules, creating a more significant number of binding sites for target biomolecules. Recent studies have demonstrated the importance of this heightened immobilization for detecting low-abundance cancer biomarkers, enabling accurate capture and measurement even at minute concentrations [31].

Another key benefit associated with the high surface-to-volume ratio in bead-based biosensors is the improved signal-to-noise ratio. The increased surface area facilitates a stronger signal from the bound biomolecules while minimizing non-specific interactions that can contribute to background noise. This specificity is vital in cancer diagnostics, where accurate identification of biomarkers amid complex biological matrices is crucial. Recent advancements in surface modification techniques have further refined the signal-to-noise ratio, thereby enhancing the reliability of bead-based biosensors in clinical applications.

The exploitation of the high surface-to-volume ratio in bead-based biosensors leads to reduced limits of detection (LODs). These biosensors can detect trace amounts of biomarkers with high precision, and thus hold promise for early cancer diagnosis. The combination of increased immobilization efficiency and reduced LODs positions bead-based biosensors as invaluable tools in identifying subtle changes in biomarker concentrations associated with early stage cancers.

#### 2.1.2. High Throughput

Bead-based biosensors offer a distinct advantage in high-throughput capabilities, allowing for the efficient and simultaneous processing of a large number of samples. This characteristic has far-reaching implications for advancing cancer research and clinical applications. The primary advantage of bead-based biosensors lies in their simultaneous multiplexed detection capability. By incorporating various bead types, each functionalized with specific capture molecules, these biosensors can detect multiple cancer biomarkers in parallel. Thus, the efficiency of diagnostic assays is enhanced, and a comprehensive overview of the patient’s disease profile in a single analysis is provided.

In addition to multiplexed detection, the high-throughput capability enables the analysis of large sample volumes within a relatively short time frame. Recent studies have demonstrated the scalability of bead-based assays, allowing high sample volumes to be analyzed without compromising the sensitivity or specificity of biomarker detection [32]. Automation and miniaturization, often coupled with high throughput in bead-based biosensors, streamline the analysis process, enabling rapid sample processing and reducing manual intervention. The outcome is minimized risk of errors. Miniaturization leads to smaller sample volumes, conserving precious clinical specimens and making these assays compatible with POC applications. These technological advancements significantly contribute to the efficiency and accessibility of bead-based biosensors in cancer-biomarker detection. Moreover, the high-throughput advantage does not come at the expense of sensitivity or specificity in bead-based biosensors. This combination of high-throughput and high-sensitivity positions bead-based biosensors as formidable tools in the early and rapid diagnosis of cancer.

#### 2.1.3. Fast Response

Bead-based biosensors have revolutionized cancer diagnostics, offering the remarkable advantage of a fast response. This characteristic is pivotal in the swift and accurate detection of cancer biomarkers, with profound implications for patient outcomes and treatment decisions.

The immediate detection of cancer biomarkers is a hallmark of the fast response advantage offered by bead-based biosensors. In clinical scenarios where time is of the essence, these biosensors have demonstrated the capability to provide actionable results within minutes. This rapid turnaround time expedites diagnosis and enables timely interventions, potentially influencing treatment strategies and improving overall patient care.

Beyond immediate detection, the fast response capability allows real-time monitoring of dynamic changes in biomarker levels. Cancer biomarkers often exhibit variations, and the ability of bead-based biosensors to capture these changes in real-time is crucial. Continuous monitoring owing to rapid response provides a dynamic picture of disease progression, offering insights into the evolving nature of cancer and its response to treatment. Early detection of cancer is a key outcome of the fast response capability of bead-based biosensors. The subtle changes in biomarker levels characteristic of early stage cancer can be swiftly identified, contributing to improving patient outcomes. The combination of fast response and high sensitivity allows these biosensors to detect low-concentration biomarkers, reinforcing their role as powerful tools in early cancer diagnosis [33].

The fast response advantage also applies to the dynamic monitoring of therapeutic responses. Changes in biomarker levels serve as indicators of treatment efficacy that can guide healthcare professionals in promptly customizing treatment plans. This real-time monitoring aspect contributes to personalized medicine approaches, optimizing patient care based on individual responses to treatment.

### 2.2. Signal-Amplification Strategies

Signal-amplification strategies are the key to enhancing the detection sensitivity of cancer biomarkers. Various approaches are utilized, including enzymatic amplification, rolling circle amplification (RCA), and hybridization chain reaction [34]. Among these technologies, ultra-sensitive probes and nucleic acid amplification contribute to increased specificity and efficiency. Additionally, concentration techniques play a crucial role in boosting signal effects. This diversified approach broadens detection capabilities and holds promise for advancing early cancer diagnosis and treatment precision.

#### 2.2.1. Ultra-Sensitive Probes

Hybridization chain reaction (HCR) is a signal-amplification strategy that has been applied to bead-based biosensors. HCR involves the sequential hybridization of short DNA hairpin probes, forming long DNA duplexes. The HCR process amplifies the signal by generating long DNA chains that can be detected using fluorescence or electrochemical methods [35]. A novel radiometric electrochemical aptasensor is developed for sensitive detection of carcinoembryonic antigen (CEA). The aptasensor utilizes a cascade reaction triggered by CEA binding to hairpin probes and subsequent Exo III cleavage, resulting in variances in oxidation peak currents of ferrocene-labeled hairpin probes and methylene blue-intercalated DNA sequences. Finally, it enables the quantification of CEA with a lower LOD of 30.5 fg/mL, demonstrating good selectivity, stability, reproducibility, and practical application in biological samples such as serum [36]. A DNA detection assay combines enzyme-free signal amplification using HCR with flow cytometry and magnetic beads for sensitive and rapid analysis. The assay immobilizes biotinylated hairpin DNA on streptavidin-functionalized magnetic beads, allowing for the target DNA to initiate a cascade of hybridization events and accumulate fluorescent signals on the magnetic beads. Flow cytometry enables quick analysis, providing quantitative results within minutes [37]. Furthermore, Kim et al. reported an enzyme-linked oligonucleotide assay that enables rapid colorimetric detection of Engrailed-2 (EN2), which is a biomarker for bladder and prostate cancer. An EN2-specific aptamer and an aptamer-mediated HCR are utilized for signal amplification, which had the advantages of high specificity and a low limit of detection in buffer and artificial urine, offering the potential for a simple, accurate, and early diagnostic tool for bladder and prostate cancers [38]. HCR amplification improves the sensitivity of bead-based biosensors and enables the detection of low-abundance cancer biomarkers.

The sensitivity of bead-based biomarker detection can also be enhanced through alternative means. An innovative biosensor using electrochemical impedance spectroscopy (EIS) has been developed. This biosensor incorporates magnetic beads confined within a microwell array, aiming to enhance the sensitivity of traditional bead-based EIS biosensors [39]. Moreover, a highly sensitive dual-signal ratio electrochemical aptasensor is exploited to construct functionalized bimetallic nanocomplexes for HER2 detection. This sensor improved the linear range of 0.75–250 pg/mL, and the LOD to 0.37 pg/mL, which provides an alternative method to detect breast cancer tumor biomarkers [40]. Recently, Janus particles (JPs) driven by rotational Brown motion have been used as novel probes for the rapid detection of diseases based on enhanced nucleic acid amplification [41] and small protein biomarkers [42]. By detecting the microviscosity change through loop-mediated isothermal amplification (LAMP) in the presence of target cDNA, SARS-CoV-2 nsp2, an LOD down to 70 ag/μL is achieved in 10 min, showcasing 100-fold higher in sensitivity and 15-fold faster than conventional polymerase chain reaction (PCR). In another study, the trace protein TNF-α is successfully measured from the decreased blinking frequency of JPs owing to their increased particle size. In this system, an LOD of 1 pg/mL is eventually obtained in 60 s.

#### 2.2.2. Nucleic Acid Amplification

RCA is a powerful signal-amplification strategy integrated into bead-based biosensors. RCA involves the circular replication of a target DNA sequence using a DNA polymerase and a circular DNA template. Each replication cycle produces a long single-stranded DNA concatemer, which can be detected using fluorescence or electrochemical tags [43]. RCA combined with specific aptamer of aflatoxin B1 (AFB1) is used to establish a highly sensitive and specific method for visual detection of AFB1. The surface of magnetic beads is coated with the AFB1 aptamer, functioning as a molecular recognition probe. The binding of the aptamer to AFB1 initiates RCA, resulting in the generation of lengthy DNA strands. These strands capture signal probes and horseradish peroxidase (HRP), inducing a significant color transformation of the solution from transparent to a deep blue hue, serving as a visual indicator for AFB1 detection. Under optimal conditions, the detection range reaches from 0.5 to 40 pg/mL and the limit of detection is 0.13 pg/mL [44]. A new self-assembly approach based on RCA enabled the ultrasensitive detection of oral cancer biomarkers micro-RNA (miRNA)21 and miRNA16, utilizing a DNA-decorated biosensor that exhibits high selectivity, wide detection range, and low limits of detection, providing a potential diagnostic tool for early stage oral cancer screening [45]. RCA amplification enhances the signal intensity and enables ultrasensitive detection of cancer biomarkers, rendering it a valuable tool in bead-based biosensors [46].

#### 2.2.3. Enrichment by Concentration

Emphasis on integrating enrichment strategies to address the challenges of identifying low-concentration or elusive biomarkers, especially in early cancer diagnosis, has grown in recent years. This shift underscores the recognition of enrichment by concentration mechanisms as a crucial tool in advancing the sensitivity, specificity, and precision of bead-based biosensors, particularly crucial in the early diagnosis and monitoring of cancer. By functionalizing microbead surfaces with specific biomolecules such as antibodies, these biosensors selectively capture cancer biomarkers. The subsequent enrichment process amplifies the concentration of biomarkers, significantly improving LODs [47,48,49]. A microfluidic-based liquid biopsy device that operates efficiently with a minimal plasma sample volume (20–50 μL), achieving a low LOD (0.1 ng/mL) and the capability to identify biomarkers swiftly within 55–75 min [50]. Chemiluminescence immunoassays also exhibit a high sensitivity and signal-to-noise ratio on cancer-biomarker detection. Fe_3_O_4_@SiO_2_ microspheres modified with Anti-CEA monoclonal antibody serve as the core for capturing CEA, whereas dendritic large-mesoporous silica nanospheres co-immobilized with anti-human CEA monoclonal antibody and HRP act as the satellite for signal amplification. These sensors demonstrate a broad detection range of 0.01−20 ng mL^−1^ and a low LOD of 3.0 pg mL^−1^, facilitating the convenient and specific CEA determination in human serum [51]. Moreover, bead-based biosensors equipped with enrichment mechanisms offer real-time monitoring of biomarker fluctuations during treatment, aiding therapy optimization [52].

#### 2.2.4. Enzymatic Amplification

Enzymatic amplification strategies have been developed to enhance the bead-based biosensors’ sensitivity and signal amplification. Enzymes can catalyze reactions that generate detectable signals, such as the conversion of a substrate into a product with a fluorescent or electrochemical signal [53]. Enzymatic amplification enables the detection of a low-concentration of cancer biomarkers by amplifying the signal through enzymatic reactions. Enzymes such as HRP and alkaline phosphatase (ALP) are extensively used in bead-based biosensors to achieve signal amplification and improve LODs [54]. For instance, a photoelectrochemical immunoassay based on magnetic beads can reportedly detect CEA specifically. Followed by magnetic separation, the HRP-labeled anti-CEA detection antibody initiates enzymatic bio-etching of hollow cadmium sulfide, decreasing photocurrent intensity. The sensing platform can work at a range from 0.02 ng/mL to 50 ng/mL and a low LOD of 6.12 pg/mL. HRP usage gives the system high precision and strong anti-interference ability [18]. Moreover, the ALP-labeled magnetic beads are used to develop an improved zinc oxide-modified carbon electrode, which is used to capture CEA in cancer samples. This system can detect CEA from 0.01 ng/mL to 6.0 ng/mL with a 4.0 pg/mL LOD. Combining magnetic beads with ALP-linked immunoassay provides a platform for rapidly and sensitively detecting biomarkers in cancer samples [55]. These signal-amplification strategies significantly improve the LODs, allowing for the more reliable and accurate diagnosis of cancer biomarkers. See Table 3.

### 2.3. Recent Trends and Innovations

**Figure 2:**
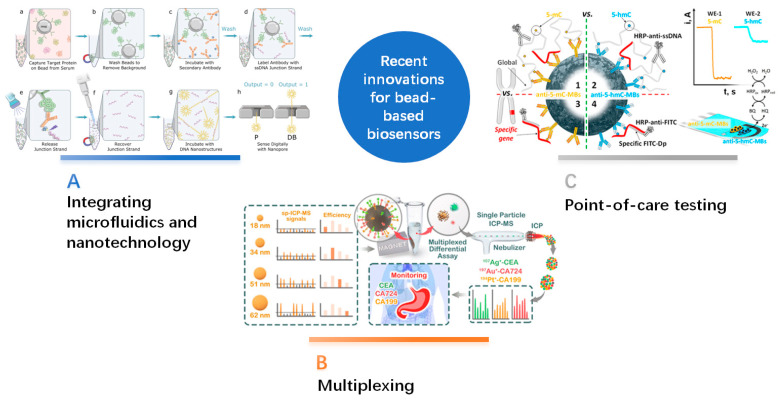
Recent innovations for bead-based biosensors. (**A**) Integrating microfluidics and nanotechnology: Digital immunoassay with nanopore electrical detection. This system shortens the analysis times and avoids non-specific signals. Reprinted with permission from [68]. Copyright 2021 Nature Communications. (**B**) Multiplexing: Single-NP differential immunoassay for multiplexed gastric cancer biomarker monitoring. This study used four different sizes of NPs to facilitate effective multiplexed assessments. Reprinted with permission from [80]. Copyright 2022 American Chemical Society. (**C**) Point-of-care testing: Merging electrochemical and microfluidic arrays enhances the potential for sensitive, high-throughput ctDNA detection, creating options for portable POCT platforms. Reprinted with permission from [86]. Copyright 2018 American Chemical Society.

Significant advancements have been made in developing bead-based biosensors for cancer-biomarker detection. These innovations aim to enhance the sensitivity, selectivity, and multiplexing capabilities of bead-based biosensors and enable integration with emerging technologies [56]. This section primarily discusses recent innovations in multiplexing, state-of-the-art POC testing (POCT) cancer diagnostics, and integrating microfluidics and nanotechnology for bead-based biosensors (Figure 2).

#### 2.3.1. Integration with Microfluidics and Nanotechnology

Microfluidic systems have been integrated with bead-based biosensors to enhance the assay performance and enable precise manipulation of samples and reagents [57]. Microfluidic platforms offer advantages such as reduced sample volumes, enhanced mixing, rapid reaction kinetics, and improved sensitivity. Microfluidic bead-based biosensors have been developed for on-chip sample processing, such as cell or virus isolation and enrichment [58], as well as multiplexed detection of cancer biomarkers. A semiconductor sensor-embedded microfluidic chip combined bead-based immunoassay with DNA strand labeling for detecting protein biomarkers has been reported. This method utilizes a magnetic bead-based immunoassay and an externally imposed magnetic force to address the issue of distance between analyte protein and the sensor surface. The use of a normal ion intensity buffer without dilution is enabled, and sensitivity is enhanced by applying longer DNA fragments and smaller magnetic beads as solid support for the antibody [59]. A rapid and simple bead-based microfluidic platform is developed for detecting a specific 22-mer DNA sequence via hybridization, utilizing single- and multi-mode interactions for probe immobilization on commercial nano-porous chromatography beads, and using a quantum dot (QD) label combined with multi-mode immobilization [60]. Moreover, an automated microfluidic platform integrated a bead-based electrochemical immunosensor with a bioreactor for continuous monitoring of cell-secreted biomarkers, it utilizes disposable magnetic microbeads for biomarker immobilization and combined microvalves into microfluidic chip to enable programmable operations of the immunoassay, allowing for its convenient integration with liver-on-chips for continual biomarker quantification [61]. From the above findings, the integration of microfluidics with bead-based biosensors facilitates the development of portable and POC devices for cancer diagnostics.

Nanoparticles (NPs) are extensively used to enhance the performance of bead-based biosensors. Functionalized NPs can be conjugated with beads or capture molecules to improve biosensors’ sensitivity, selectivity, and signal amplification [62]. For example, magnetic NPs can be used for efficient target capture and separation, whereas plasmonic NPs can enhance the signal readout through enhanced fluorescence or Raman scattering [63]. A high-sensitivity bead-based immunoassay with nanofluidic preconcentration has been reported for biomarker detection. It exploits an antibody-coated bead-based immunosensor in a valve-integrated nanofluidic preconcentration device, where concentrated antigens and antibody-coated nanobeads are isolated and the antigen concentration can be determined in real time through micro-particle-tracking velocimetry. This method can rapidly detect PSA with a 50 pg/mL LOD in only 20 min [64]. Thus, NP-enhanced bead-based biosensors offer improved LODs, increased specificity, and the potential for multiplexed detection of cancer biomarkers.

Nanopore-based biosensors have emerged as a promising approach for the detection of cancer biomarkers [65]. Nanopores are nanoscale pores that allow the passage of biomolecules through them. By functionalizing beads with capture molecules and introducing them into a nanopore system, the binding events between the target biomarker and the capture molecule can be detected based on changes in ionic current or impedance [66]. An electrical biosensing method is established using synthetic nanopores and nanochannels integrated into fluidic devices for versatile analyte detection through size calibrations. The method is a low-complexity, low-cost fabrication technique suitable for industry production [67]. In addition, a digital immunoassay utilized solid-state nanopores for accurate quantification of biomarker concentrations, which addressed the challenges of specificity, sensitivity, and consistency by using identifiable DNA nanostructures to represent the presence or absence of the target protein [68]. Chuah et al. developed a nanopore blockade sensor approach for highly sensitive protein detection in complex biological samples, which utilized antibody-modified magnetic NPs to capture the analyte. By immune-sandwich formation in the nanopore, this system shortens the analysis times and avoids non-specific signals, indicating its potential for quantitative analysis of diverse protein and nucleic acid species [69]. Nanopore-based biosensors offer label-free, real-time detection, high sensitivity, and the potential for single-molecule analysis [70]. Moreover, an alternative exosome isolation method [71], known as the Exosome Total Isolation Chip (ExoTIC), has also been reported. This novel size-based platform based on a polycarbonate track-etched nanoporous filter membrane allows the efficient and standardized purification of extracellular vesicles (EVs) from different biofluids. The ExoTIC system is notable for its modular design, which facilitates the size-exclusive sorting of heterogeneous EV subpopulations. In contrast to traditional ultracentrifugation techniques, ExoTIC demonstrates a significant 4- to 1000-fold increase in EV yield. Furthermore, its capability to isolate EVs effectively from small sample volumes ranging from 10 to 100 μL makes it particularly well-suited for preclinical investigations involving small animal tumor models and point-of-care clinical applications that rely on capillary blood samples acquired via finger pricks.

#### 2.3.2. Multiplexing

Standard immunoassays are limited in their ability to detect only one specific analyte, which is inadequate for accurate early diagnosis under conditions like cancer where multiple biomarkers are involved. Consequently, the use of multiple immunoassays becomes necessary. However, these immunoassays require meticulous collection, labeling, storage, and banking of samples according to strict laboratory protocols, and are thus challenging. Achieving early disease diagnosis depends on extracting the maximum information from minimal clinical samples. In such scenarios, multiplexed immunoassays emerge as a compelling solution particularly when dealing with limited sample volumes. The primary advantage of multiplexed immunoassays is their ability to detect multiple biomarkers qualitatively or quantitatively within a single sample. This method offers several benefits, including increased data points per sample, reduced cost per data point, fewer errors owing to fewer samples, and improved efficiency. However, a significant challenge remains in cross-reactivity, thereby reducing the specificity of immunoreactivity and hindering the proper functionality of multiplexed detection in complex biological and clinical samples.

Multiplex magnetic bead assays are widely utilized to analyze multiple substances in clinical samples. The most commonly used method among them is the Luminex assay, which combines an enzyme-linked immunosorbent assay (ELISA) with flow cytometry [72]. In this method, paramagnetic microspheres labeled with different fluorophores are conjugated to specific capture antibodies for the analytes. These beads bind to analytes in the sample, resulting in the formation of an antibody-antigen sandwich structure upon addition of biotinylated detection antibodies. The introduction of fluorophore-labeled streptavidin enables detection using a dual-laser flow-based instrument, thereby facilitating the identification and quantification of multiple biomarkers with precision in biofluid samples. Alternatively, magnetic beads and QDs can be combined for detection, which is known as the magnetic bead–QD assay. Researchers have explored various methods for detecting lung cancer biomarkers. Liu et al. developed a multiplex magnetic bead–QD assay in a microarray format to detect the biomarkers CYRFA 21-1, neuron-specific enolase (NSE), and CEA associated with lung cancer. They utilized magnetic beads and QDs conjugated with specific antibodies in serum samples, successfully detecting these biomarkers even at low concentrations (LOD: 364 pg/mL for CYRFA 21-1, 38 pg/mL for CEA, and 370 pg/mL for NSE) [73,74]. In another study, Bai et al. used a bead-based microarray to detect the lung cancer biomarkers CEA, CYFRA 21-1, and ProGRP from exosomes [75]. They collected exosomes using a microfluidic system from samples of lung cancer patients and used QDs conjugated with detection antibodies to detect the tumor biomarkers. The results of their method exhibit minimal differences from clinical data, suggesting its potential applicability in clinical testing. Additionally, Li and collaborators developed barcodes for multiplexed detection using magnetic beads and QDs [76]. They introduced barcodes capable of detecting five tumor biomarkers simultaneously in serum samples, achieving detection even at sub-ng/mL concentrations.

QD-linked immunosorbent assay (QLISA) is a variant of the ELISA that uses QDs instead of enzymes. QDs act as amplifiers, enabling the detection of low-concentration analytes. Owing to their optical and chemical properties, QDs are well-suited for multiplexing. QDs also can be used in multiplexed electrochemical immunoassay. Guo et al. developed a novel multiplex electrochemiluminescence immunoassay for the simultaneous detection of two tumor biomarkers (AFP and CEA) in human serum and saliva [77]. This method used dual-color QD labels (525 nm and 625 nm) in conjunction with graphene as a conductive bridge. Streptavidin-coated CdSe/ZnS QDs, along with biotin-labeled secondary antibodies specific to AFP and CEA, are used to induce electrochemiluminescence reactions following the immunoreaction. The addition of graphene significantly enhanced the intensity of the electrochemiluminescence signal. Quantification of AFP and CEA levels is achieved by measuring the electrochemiluminescence responses of QDs525 and QDs625, respectively. The assay demonstrated a broad working range spanning from 0.001 to 0.1 pg/mL and a LOD of 0.4 fg/mL for both biomarkers. In a study conducted by Aneta et al. presented a novel magneto-immunosensor that utilized electrochemical nanocomposites for the simultaneous quantification of three ovarian cancer biomarkers (human epididymis secretory protein 4 (HE4), AFP, and CA-125) [78]. The sensor incorporated three distinct electroactive nanomaterials, including gold NPs (AuNPs), CdTe, and PbS core QDs, each conjugated with specific antibodies. The addition of mesoporous silica NPs (SiNPs) in the nanocomposite enhanced the electrochemical signal through increased label loading.

AuNPs are non-toxic and biocompatible. Thus, they are commonly used in the research and development of diagnostic tools, drug-delivery systems, novel therapeutics, and other medical applications. Among the important methods for detecting biomolecules such as DNA, RNA, enzymes, proteins, and small molecules are NP-based colorimetric assays. The ability to change the color of a colloidal solution owing to variations in the size or distance between noble metal NPs is a crucial factor in colorimetric sensing. Colorimetric sensors have several types, namely, aggregation, etching, growth, and nanoenzyme. Di et al. demonstrated various techniques for multiplexed colorimetric cancer detection in their study [79]. They used decorated AuNPs and antibody-conjugated exosomes in a nanozyme-assisted immunosorbent assay to detect exosomal proteins CD63, CEA, GPC-3, PD-L1, and HER2 from cell lines and clinical serum samples. This approach allows for the differentiation of protein levels without the need for additional labeling with detection antibodies, thereby providing a faster and simpler testing procedure. Huang et al. present a new wash-free immunoassay called the “differential assay”, which utilizes single-particle inductively coupled plasma mass spectrometry to quantify unbound NP tags on a solid support [80]. To facilitate effective multiplexed assessments, the researchers produced NPs with four different sizes and determined the optimal size. The wash-free approach is successfully implemented for concurrent evaluation of serological biomarkers (CA724, CA199, and CEA), yielding results that align with established clinical methods.

In sum, recent advancements in bead-based biosensors have been primarily centered around enhancing fabrication techniques, signal-amplification strategies, and the amalgamation of microfluidics and nanotechnology. These advancements significantly augment the sensitivity, selectivity, and multiplexing capabilities of bead-based biosensors for detecting cancer biomarkers. Integrating emerging technologies with bead-based biosensors tremendously increases the potential to transform cancer diagnostics. It can facilitate the creation of swift, sensitive, and portable devices for early prognostication and therapy monitoring. Continued research and development in this domain are expected to further propel the functionalities of bead-based biosensors and pave the way for their extensive adoption in clinical settings.

#### 2.3.3. State-of-the-Art POC Cancer Diagnostics

The development of POCT cancer diagnostics has revolutionized the field of cancer detection, providing more effective and accessible options for patients. These advanced technologies aim to bring the testing and evaluation of cancer biomarkers closer to patients. Accordingly, the time and resources required for diagnosis are reduced. By bridging the gap between laboratory-based tests and bedside or community healthcare settings, POCT diagnostics deliver real-time results, enabling faster clinical decisions. Rapid detection is a defining characteristic of POCT cancer diagnostics. These systems are designed to provide results within minutes, helping healthcare professionals make immediate decisions. In the case of cancer, early diagnosis is crucial. Thus, rapid detection can significantly improve patient outcomes.

The miniaturization and portability of the state-of-the-art POCT devices render them highly user friendly and accessible. These compact and portable devices are ideal for use in resource-limited settings, remote areas, and even at-home testing. The miniaturization of technology has also facilitated the development of handheld or smartphone-linked POCT devices, further increasing accessibility for patients and healthcare providers. Another important feature of POCT cancer diagnostics is their high sensitivity and specificity. Sensitivity ensures the detection of even small amounts of cancer biomarkers, whereas specificity ensures the test does not produce false-positive results. Advances in nanotechnology, microfluidics, and biotechnology have contributed to improving the accuracy of POCT devices [81]. Many state-of-the-art POCT devices have multiplexing capabilities, enabling the simultaneous detection of multiple cancer biomarkers [82,83]. This is particularly valuable because cancer often exhibits complex molecular profiles. POCT diagnostics can provide a more comprehensive view of the disease by detecting a combination of biomarkers.

Bead-based biosensors play a crucial role in POCT cancer diagnostics. These biosensors utilize micro- or nanoscale beads, functionalized with specific antibodies or aptamers, to capture and detect cancer biomarkers. Bead-based biosensors offer a high surface area for biomarker binding, enhancing sensitivity. Additionally, they can be integrated into microfluidic platforms, enabling efficient sample handling and analysis. Their adaptability to various cancer biomarkers makes them versatile for POCT applications. Affordability is another essential aspect of POCT diagnostics. These devices are designed to be cost effective, reducing the need for expensive laboratory equipment and specialized personnel. This makes them suitable for widespread adoption, particularly in healthcare systems with limited resources. Some POCT devices offer real-time monitoring capabilities, allowing for continuous tracking of cancer biomarkers. This feature is particularly valuable in assessing treatment responses and disease progression, offering a dynamic approach to cancer management. Many state-of-the-art POCT cancer diagnostics have wireless connectivity, enabling data transmission to healthcare providers or electronic health records. This connectivity enhances the sharing of diagnostic information and facilitates remote consultations, ultimately improving patient care.

The development of state-of-the-art POCT cancer diagnostics has led to significant advancements in cancer detection. These devices offer rapid detection, miniaturization, high sensitivity, multiplexing capabilities, bead-based biosensors, cost effectiveness, real-time monitoring, and wireless connectivity [84,85]. With the potential to revolutionize cancer diagnostics, POCT devices can improve patient outcomes and make cancer screening more accessible.

### 2.4. Materials for Bead-Based Biosensors

**Figure 3:**
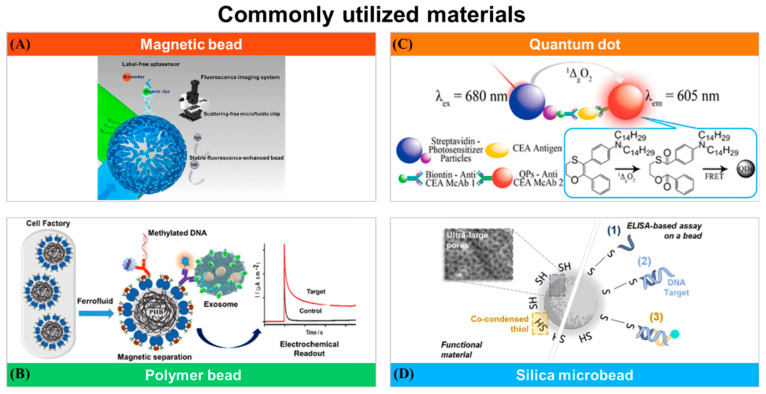
Commonly utilized materials in bead-based biosensors. (**A**) This study establishes a movable magnetic bead-based biosensing platform that combines fluorescence enhancement and a microfluidic chip, overcoming the limitations of fixed substrates and demonstrating improved signal-to-noise ratio by using zinc oxide nanorod-decorated magnetic beads. Reprinted with permission from [87]. Copyright 2023 American Chemical Society. (**B**) The bioengineered multifunctional core–shell structures comprise a poly-3-hydroxybutyrate core densely coated with protein functions for use in bioseparation and immunodiagnostic applications. Reprinted with permission from [93]. Copyright 2021 American Chemical Society. (**C**) Quantum dots (QDs) present favorable photophysical properties. In this work, a chemiluminescent homogeneous-detecting biosensor is fabricated using QD-doped polystyrene nanospheres to detect biomarkers in low-volume serum samples sensitively. Reprinted with permission from [97]. Copyright 2019 American Chemical Society. (**D**) This study focuses on synthesizing large-pore silica microbeads to serve as 3D capturing structures, optimizing a bead-based bioaffinity assay. Using an ELISA-like approach, the mesoporous system effectively functions as the dispersed detection phase, demonstrating its capability to accurately measure DNA concentration within the range of 0–1 nM. Reprinted with permission from [102]. Copyright 2020 American Chemical Society.

The choice of materials greatly affects the development and efficacy of bead-based biosensors in various applications, such as cancer detection. These materials serve as the fundamental base for constructing biosensors and directly impact their sensitivity, specificity, and overall performance. This discussion explores the commonly utilized materials in bead-based biosensors and their vital role in advancing these innovative diagnostic tools (Figure 3).

#### 2.4.1. Magnetic Beads

Magnetic beads, often composed of materials like iron oxide, bring the advantage of easy manipulation through external magnetic fields. This property facilitates rapid and efficient separation, enhancing the overall sensitivity and speed of biosensing applications. Meeseepong et al. established a movable magnetic bead-based biosensing platform, thereby overcoming the limitations of fixed substrates, integrating digital imaging, and demonstrating improved signal-to-noise ratio using zinc oxide nanorod-decorated magnetic beads. The platform combines fluorescence enhancement and a microfluidic chip, allowing continuous biomarker detection and offering potential applications in diagnostics and biological assays [87]. Self-assembly techniques are eliciting increased attention in the fabrication of bead-based biosensors. Self-assembly involves spontaneously organizing beads into well-defined patterns or structures through non-covalent interactions. By controlling the beads’ size, shape, and composition, self-assembly techniques enable the fabrication of highly ordered arrays or hierarchical structures, which can enhance the sensitivity and reproducibility of the biosensors [88]. Magnetic beads made of iron oxide NP-embedded polymer matrices can self-assemble at specific locations on functionalized surfaces [89]. Yue et al. also used a magnetically induced self-assembly technology to establish a label-free electrochemical biosensor on a magnetic nanocomposite to detect biomarker CA125 [90]. Self-assembly has been used to create 2D and 3D bead arrays, enabling high-density immobilization of capture molecules and improved detection performance. Magnetic beads play a pivotal role in magnetic biosensors are extensively used in biomolecule separation, cell isolation, and magnetic bead-based assays.

#### 2.4.2. Polymer Beads

Polymeric materials offer excellent biocompatibility and tunable properties. For instance, hydrogels or microspheres can be engineered to encapsulate various biomolecules, which provide a stable and protective environment for sensitive biological elements. A glass-polymer biosensor utilizes distinct beads with oligonucleotide probes, sequentially spotted on gel pads, enabling immobilization and identification without encoding. This approach enhances throughput for DNA-based detection of single-nucleotide polymorphisms with single-mismatch specificity in under 10 min through passive hybridization [91]. Moreover, hydrogel-crosslinked hydrophilic polymers available in natural and synthetic forms, they exhibit biocompatibility, sustainability, sensitivity, and control release activated by magnetic or fluorescent modifications, necessitating the ongoing development of crosslinked polymers [92]. Moreover, a multifunctional nanobead is established via the self-assembly of poly-3-hydroxybutyrate (PHB) with a ferritin-derived iron-binding peptide and a protein A-derived antibody-binding Z domain. The outcome is efficient magnetic separation for enhanced electrochemical detection of cancer biomarkers, such as methylated DNA and exosomes from cancer cells. Accordingly, a foundation for the development of advanced nanomaterials for diverse and sensitive diagnostic applications is established [93]. Polymer beads can be applied in controlled drug delivery and enzyme immobilization. They can also serve as versatile carriers in biosensing platforms.

#### 2.4.3. Quantum Dots

QDs are nanoscale semiconductor particles exhibiting unique optical or electronic properties. Their size-tunable emission spectra and high photostability make them valuable in fluorescence-based biosensors, allowing for precise and multiplexed detection. Fluorescent QDs, with a diameter <10 nm, represent a cornerstone in nanoscience and nanotechnology, offering unique structural, electrochemical, and photochemical properties that make them a promising platform for biosensing. QDs are characterized by high potential for signal enhancement, high functionalization capacity with bioreceptors, and the ability to integrate nanotechnology and biotechnology position. Thus, QDs are key elements in advancing electrochemical biosensors for the early diagnosis of diseases, including tumor markers, inflammatory biomarkers, depression markers, and diabetes-related biomolecules [94]. A rapid electrochemical magnetic immune-sensing utilizes carboxylic acid-functionalized magnetic beads and core/shell streptavidin-modified CdSe@ZnS QDs. It demonstrates a linear detection range of 0.50–50 ng/mL and an LOD of 0.29 ng/mL for the breast cancer biomarker HER2-ECD. The assay exhibits excellent selectivity for the HER2-positive breast cancer cell line SK-BR-3 with a concentration-dependent signal 12.5× higher than HER2-negative cells (MDA-MB-231) [95]. Utilizing QDs and magnetic NPs, the magnetic QD microbeads are used as dual-functional carriers for optical encoding and magnetic separation. The introduced catalytic hairpin assembly and terminal deoxynucleotidyl transferase (CHA-TdT) cascade amplification enables the detection of bladder cancer-related miRNAs in clinical serum specimens with femtomolar sensitivity, a wide linear dynamic range, and consistency with qRT-PCR, showcasing potential for multiplexed miRNA detection in the clinical diagnosis and early detection of bladder cancer [96]. A chemiluminescent homogeneous biosensor has been reported to utilize QD-doped polystyrene nanospheres. It demonstrates exceptional sensitivity, achieving an unprecedented LOD of 2.56 × 10^–13^ M (46 pg/mL) for CEA in 25 μL serum samples, with strong correlations (R^2^ = 0.99718, n = 10^7^) observed between this biosensor and commercial chemiluminescence immunoassay kits in clinical serum detection. This approach is promising for early disease detection and prognosis evaluation [97]. A novel QD nanobead-based fluorescence-linked immunosorbent assay platform has been developed for highly sensitive multiplexed detection of lung cancer biomarkers, achieving a 100-fold improvement in detection sensitivity compared with conventional approaches, and demonstrating consistency with the clinical gold-standard electrochemiluminescence immunoassay (ECLIA) in human serum samples [98]. QDs are extensively used in cellular imaging, medical diagnostics, and as labels in bioassays, expanding the capabilities of optical biosensing.

#### 2.4.4. Gold Nanoparticles

AuNPs possess exceptional optical properties, including surface plasmon resonance (SPR), which contributes to their remarkable sensitivity in colorimetric biosensing. Their ease of functionalization allows for the attachment of various biomolecules, enhancing specificity. AuNPs, renowned for their exceptional physical and chemical properties, have found extensive application in the development of biosensing strategies. AuNP-based biosensing has been widely applied for detecting tumor-related biomarkers in bodily fluids, encompassing optical, electrochemical, and mass spectrometric approaches known for their outstanding performance in tumor biomarker assays [99,100]. Commonly utilized in colorimetric assays, immunoassays, and DNA sensing, AuNPs have become integral components in diverse biosensing strategies.

#### 2.4.5. Silica Beads

Silica beads offer a stable and inert matrix, providing an ideal support for the immobilization of biomolecules. The porous structure allows for high loading capacity and efficient diffusion of target analytes. Bu et al. developed a highly sensitive circulating cell-free DNA (cfDNA) capture system using polydopamine (PDA) and silica, which increases the capture efficiency by 1.34-fold compared with conventional silica-based methods. In clinical samples, this system shows superior diagnostic accuracy over commercial cfDNA kits and serum antigen tests, correlating well with tumor size and predicting distant metastasis. Additionally, this technology exhibits high concordance with tissue biopsy results, particularly in detecting HER2-positive tumors [101]. By using monoclonal antibody-coated polystyrene nanobeads, an ultra-sensitive biosensor has been assembled in microchip trenches. It enables the detection of cancer biomarkers, specifically nucleosomes and CEA, at concentrations as low as 62.5 and 15.6 pg/mL, respectively [23]. Ultralarge-pore silica microbeads are synthesized via a one-pot method with optimized condensation, this microbeads can be used as a versatile three-dimensional (3D) substrate for the development of an ELISA-like DNA detection, exhibiting superior bead-capturing ability and a two-fold lower LOD compared with a standard flat surface assay or traditional ELISA [102]. This kind of silica beads offer a stable and compatible surface for biomolecule immobilization and are used in various biosensing platforms for cancer-biomarker detection.

#### 2.4.6. Luminescent/Fluorescent Beads

Polystyrene or similar polymers containing embedded luminescent or fluorescent dyes are engineered into microbeads to emit light upon excitation, enabling optical detection and compatibility with fluorescence-based detection methods. A novel conceptual approach for enhancing bead-supported assays introduces optical tweezers to enable precise imaging. The strategy uses luminescence-confined NPs with a unique sandwich structure for efficient energy transfer, achieving a high luminescent resonance energy transfer ratio to FAM molecules. This approach enables miRNA analysis with a sub-femtometer LOD, and low-abundance targets as few as 30 cancer cells can be accurately qualified. Thus, this technique can serve as a valid cancer early warning tool for liquid biopsy [103]. Li et al. introduces a bead-based assay by using holographic optical tweezers and upconversion luminescence encoding, achieving stable excitation conditions for specifically detecting two liver cancer-related biomarkers, including CEA and alpha-fetoprotein [104].

In conclusion, the materials selected for bead-based biosensors are pivotal in determining their effectiveness in cancer detection and other applications. The selection of bead type in biosensors is a crucial decision that relies on the specific requirements of the intended application. Each type of bead possesses distinct mechanical, optical, or magnetic properties, enabling the customization of biosensors to cater to the demands of diverse sensing environments. With the continuous advancement of the field, the incorporation of innovative materials and the enhancement of existing bead-based biosensor technologies hold the potential to unlock novel possibilities for achieving highly sensitive and specific detection in various disciplines.

### 2.5. Biosensing Technologies

**Figure 4:**
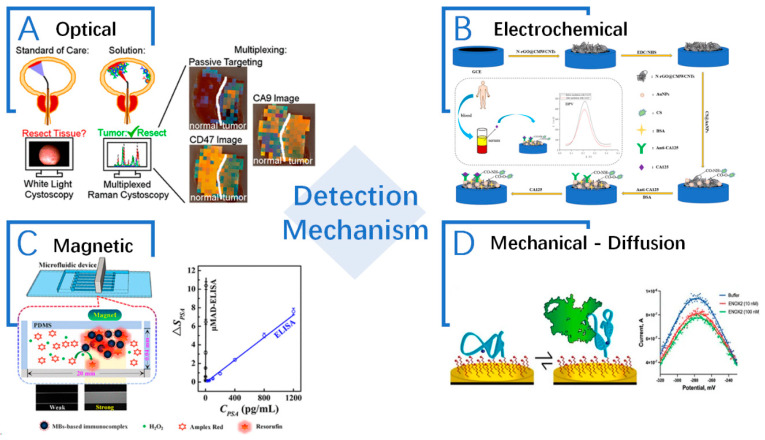
Types of bead-based biosensors by detection mechanism. (**A**) Surface-enhanced Raman scattering (SERS) NPs for multiplexed imaging of bladder cancer-biomarker detection. Reprinted with permission from [108]. Copyright 2018 American Chemical Society. (**B**) Electrochemical immunoassay for tumor marker CA125. Reprinted with permission from [109]. Copyright 2022 by the authors. Creative Commons Attribution (CC BY) license. (**C**) Analytes are identified and separated from a complex sample matrix by using magnetic beads and then concentrating them in the magnetic trapping/detection area for direct in situ fluorescence detection. Reprinted with permission from [110]. Copyright 2021 American Chemical Society. (**D**) In the E-DNA biosensor platform for early cancer detection, alterations in molecular dynamics, such as size, flexibility, and diffusion rate, induce a conformational shift in aptamers, leading to a detectable shift in the current readout. ENOX2 is shown in green, biosensor in light blue, and methylene blue is shown as a dark blue sphere. Reprinted with permission from [111]. Copyright by the authors. Licensee MDPI, Basel, Switzerland. (CC BY 4.0).

Bead-based biosensors offer a versatile platform for the detection of cancer biomarkers by utilizing microscopic beads as a solid support. These beads can be functionalized with specific capture molecules, such as antibodies [105], aptamers [106], or nucleic acids [107], enabling the selective binding and detection of target biomolecules. Bead-based biosensors are classified into different types based on the detection mechanism used. Generally, bead-based biosensors have four main types, namely, optical, electrochemical, magnetic, and mechanical bead-based biosensors (Figure 4).

#### 2.5.1. Optoelectrical Biosensors

Optical bead-based biosensors utilize various optical principles to detect and quantify the binding events between the target biomarker and the bead-bound capture molecule [112]. Some of the commonly used optical techniques in bead-based biosensors include fluorescence, SPR, and Raman spectroscopy.

Fluorescent bead-based biosensors utilize fluorescent labels on the beads to enable sensitive and quantitative detection. The beads are excited with a light source, and the emitted fluorescence signal is measured using a fluorescence microscope or a flow cytometer. The strength of the fluorescence signal is correlated with the amount of target biomarker bound to the beads, allowing for quantification [87]. The dual-capturing antibodies against HER2 and CA125 are incorporated into a single three-dimensional porous calcium alginate bead for differentiating HER2 and CA125 in serum samples from breast cancer patients [113]. Utilizing a composite of Ag2S QDs and CA125 aptamer-combined 5-fluorouracil, the probe is used to achieve near-infrared photoluminescence turn-on detection of the CA125 antigen [114]. An antibody–single-strand DNA (ssDNA) aptamer sandwich-type fluorescence immunosensor is developed based on aptamer-functionalized carbon dots and CA125 antibody-attached PAMAM–dendrimers/AuNPs. The fluorescence resonance energy transfer (FRET) signals between carbon dots and AuNPs is related to CA125 concentrations [115]. Similarly, the FRET signals between DNA-conjugated CdTe QDs and fluorescent labeled 7-amino-4-methylcoumarin-3-acetic acid (AMCA) can reflect the glycans of Mucin 1 on cancer cell surface [116]. The biotin-labeled Muc1 aptamer can link the streptavidin-modified magnetic microparticles, and then it is used to measure the fluorescence of the CdZnTeS QDs in serum samples through specifically capturing Muc1 antigen [117]. Fluorescent bead-based biosensors offer high sensitivity, multiplexing capabilities, and compatibility with different fluorescent dyes, making them widely used in cancer-biomarker detection.

SPR bead-based biosensors measure the variance in refractive index on the bead surface upon biomolecular binding [118]. When the target biomarker binds to the capture molecule on the beads, it induces a change in the local refractive index and a shift of SPR angle. This shift is then measured and correlated with the concentration of the target biomarker [119]. For instance, CA125 in serum is anchored onto a gold rod electrode. The percentage of 11-mercaptoundecanoic acid coverage on the electrode surface is measured using SPR to reflect the CA125 concentration [120]. In cancer-positive samples, the immunoreaction between the gold-nanorod-modified CA125 antibody and CA125 antigen occurs in an aqueous solution. Meanwhile, CA125 antigen quantity is reflected by the level of gold-nanorod aggregation, which actually originates from CA 125 antibody-antigen interactions using SPR [121]. A novel localized SPR biosensor is also developed for recognizing the HE4 biomarker from ovarian cancer patients, by which the anti-HE4 antibody is used as a probe and assembled on the surface of nanochip to distinctly recognize the HE4 antigen [122]. SPR bead-based biosensors offer real-time monitoring and label-free and high sensitivity detection, making them suitable for the detection of cancer biomarkers.

Raman spectroscopy bead-based biosensors utilize the Raman scattering phenomenon to detect the target biomarker. The beads are functionalized with the captured molecules. When the target biomarker then binds to the beads, it induces changes in the Raman scattering signal. By analyzing the Raman spectra, the presence and concentration of the target biomarker can be determined [123]. The surface enhanced Raman scattering (SERS) is used for detection of the breast cancer biomarker MUC1, and the MUC1 specific aptamer is fabricated on core–shell (Au@Fe_3_O_4_) NPs to capture MUC1 molecules on the surface of tumor cells [124]. Furthermore, a PCR-SERS is developed to detect the mutations of V600E at B-Raf oncogene gene and E542K at phosphatidylinositol 3-kinase catalytic subunit alpha (PIK3CA) gene, which are closely associated with right-sided colon cancer [125]. Additionally, an improved SERS with fabricating Raman reporters to silver NP films (AgNFs), so the DNA probes for a MicroRNA 223 (miR-223) and an α-Fetoprotein (AFP) antibody can be covalently bound to Raman reporter domains for the detection of miR-223 and AFP, which are specific biomarkers of liver cancer [126]. For human bladder cancer samples diagnosis, the SERS NPs endoscope system is designed to trace CD47 and Carbonic Anhydrase 9 in tumor tissues via ex vivo imaging [108]. A dual-SERS biosensor with Fe_3_O_4_@Ag-DNA-Au@Ag@DTNB (SERS tag) conjugates is suitable for microRNA-10b (miR-10b) detection in exosome and plasma samples, in which miR-10b is recognized using DNA probes and the SERS tag is released to trigger intensity quenching [127]. Raman spectroscopy-based bead-based biosensors offer high specificity, multiplexing capabilities, and compatibility with different Raman-active labels, enabling the sensitive and selective detection of cancer biomarkers [128]. See Table 4.

#### 2.5.2. Electrochemical

Electrochemical bead-based biosensors rely on the measurement of electrical signals resulting from the redox reactions associated with the binding events on the bead surface. These biosensors are sensitive, rapid, and compatible with portable and miniaturized devices [27]. Electrochemical deposition techniques are used for the controlled and precise fabrication of bead-based biosensors. These techniques involve the electrodeposition of materials onto the bead surface, allowing for the formation of functional layers with desired properties [129]. For example, metal or metal-oxide layers can be deposited to improve biosensors’ conductivity, stability, and sensitivity [130]. An electrochemical nano-biosensor-immobilized aptamer chain is fabricated on a glassy carbon electrode’s surface to detect the cancer biomarker miRNA-128 [131]. Electrochemical deposition techniques enable the fabrication of uniform and well-defined coatings on the bead surface, facilitating the efficient and reliable detection of cancer biomarkers.

Electrochemical bead-based biosensors are extensively exploited in breast cancer detection. The dye-labeled DNA probe is used to fabricate the electrode, and CA15-3 ranging within 0.01–1 U/mL is the target for detection [10]. Breast cancer type 1 susceptibility protein (BRCA1) protects DNA replication forks and facilitates DNA double-strand break repair. BRCA1 is usually taken as the target biomarker for detecting breast cancer [132,133]. EGFR plays a crucial role in cell growth, division, and survival [134]. In cancer cells, the activated EGFR signaling pathway promotes uncontrolled cell growth, invasion, and resistance to cell death, contributing to tumor formation and progression [135]. EGFR is anchored onto the cell surface and is also a target biomarker for breast cancer diagnosis [136]. In addition, the DNA biosensors utilizing AuNP-modified graphene oxide have been developed for the early diagnosis for breast cancer [137].

Some lung cancer detections are also dependent on electrochemical bead-based biosensors. The DNA probe is a popular method for lung cancer detection, for example, the ssDNA modified probe for CYFRA21-1 gene [11], ssDNA λ-exon-modified probe for EGFR [13], and primer probe for human maternally expressed gene 3 [12] have been successfully developed for lung cancer detection. The antibody-antigen interaction is used to construct a sandwich-type immunoassay for lung cancer detection [138]. A sandwich-type electrochemical immunoassay is fabricated with three-dimensional graphene and glutaraldehyde onto the carbon electrode. This method is specific for detecting lung cancer biomarker CYFRA21-1 [139].

Electrochemical bead-based biosensors are also used for prostate cancer diagnosis. Moon et al. exploited PSA as the target biomarker, and the PSA antibody is directly incorporated into a three-dimensional Au nanowire array with electropolymerized poly-pyrrole [19]. miR-21 has become a new reliable biomarker candidate for cancer detection. Dendritic gold nanostructures functionalized with thiolate acceptor probes are grafted onto single-walled carbon nanotubes on the surface of fluorine-doped tin oxide. Then, cadmium ion-labeled miR-21 target is taken as the signal-amplification substance to specifically recognize miR-21 [140].

For ovarian cancer diagnosis, electrochemical bead-based biosensors are also developed to detect CA125, which is a cancer antigen and known as the top biomarker [141]. An electropolymerized polyaniline layer is applied onto a graphene screen-printed electrode. To enhance its functionality for ovarian cancer diagnosis, the sensor surface is further cross-linked with anti-CA125 antibodies [25]. Similarly, The Mucin-16 antibody is immobilized at the bio-interface of graphene QD ink (GQD) to sensitively recognize low-concentration CA-125 biomarker in human plasma samples [142]. Moreover, the CA125 antibodies are directly immobilized onto Au-Ag NPs to form the immunosensor for a linear response of cancer antigen CA125 [143].

Some universal targets are also developed as biomarkers for cancers diagnosis. The novel iron nitride NPs are subjected to nitridation, and the nanocomposite-modified screen-printed carbon electrode can perfectly sense 4-nitroquinoline N-oxide (4-NQO), which is a vital biomarkers for cancers [144]. The protein p53 is a common tumor-suppressor gene and plays an inevitable role in proliferation and apoptosis, so it is widely taken as a cancer biomarker. CdS nanocrystals are immobilized on the carbon electrode to form a sandwich-type immunocomplex with AuNPs, which is used to detect p53 in cancer cells [145]. To establish an exceptionally functional surface, core–shell nanofibers [146], multi-walled carbon nanotubes [109] and AuNPs [147] are used to modify a glassy carbon electrode. On this modified electrode, a tumor marker MUC1-binding aptamer is fabricated, allowing for specific detection and analysis. The MUC1 concentration can be measured using electrochemical impedance spectroscopy according to the resistance change of the electrode surface [148]. In addition, similar cancer biomarkers, including (CEA) [149], sex-determining region Y-box 2 (SOX2) [150], multidrug resistance (MDR) [151], and oxidative stress biomarker 8-hydroxydeoxyguanosine (8-OhdG) [152] play a remarkable role in the progression of various cancers, and they are already developed as the targets for cancers diagnosis. A GQD composite is formed by fabricating GQDs to an enzyme-free electrochemical immunosensor towards CEA [149]. The anti-SOX2 antibody is conjugated to indium tin oxide-based electrode for biosensing the interaction with SOX2 antigen [150]. A modified electrode incorporating AuNPs and toluidine blue-graphene oxide is used as a platform for a label-free electrochemical DNA biosensor. This biosensor is designed to accurately detect and quantify the MDR1 gene [151]. The antibodies against 8-OhdG are fabricated to the surface of a silicon nanowire-based biosensor for early cancer diagnosis [152]. All electrochemical biosensors above are listed in Table 5.

#### 2.5.3. Magnetic Biosensors

Magnetic bead-based biosensors utilize the magnetic properties of beads for the detection of target biomarkers. These biosensors offer advantages such as rapid and efficient target capture, easy separation, and compatibility with miniaturized devices. Magnetic bead-based biosensors are widely used in cancer-biomarker diagnosis [153].

Giant magnetoresistance (GMR) bead-based biosensors utilize the changes in resistance resulting from the binding events between the target biomarker and the bead-bound capture molecule. The beads are functionalized with capture molecules, and when the target biomarker binds, it induces changes in the magnetic field around the GMR sensor, leading to changes in resistance [154]. The resistance change is then measured and correlated with the concentration of the target biomarker. For example, three lung cancer biomarkers including CEA, CYFRA21-1, and NSE are monitored using bead-based chips within the polydimethylsiloxane (PDMS) chamber, which consists of a sandwich structure of magnetic beads and the QD probes [73]. An innovative Suspension Magnetic-Bead-based Assay (SUMBA) is created for identifying the potential cancer biomarker known as aberrant glycans (AGA). This method is optimized through energy dispersive X-ray spectroscopy and SPR analyses [155]. Magnetic bead-based electrochemical and colorimetric methods can also reportedly detect the sugar units on cancer cell surface, which can be recognized by aptamer modified magnetic beads, then sequestrated ferroceneboronic acid or 4-mercaptophenylboronic acid to decrease the electrochemical signal [156]. These GMR bead-based biosensors offer high sensitivity, multiplexing capabilities, and compatibility with portable devices.

Moreover, a photoelectrochemical immunoassay based on magnetic beads has been reported to specifically detect CEA. The hollow cadmium sulfide serves as photoactive matrix on immunomagnetic separation. This method demonstrates remarkable precision, strong resistance to interference ability, and acceptable accuracy by combining with magnetic immunoassay [18]. Similarly, using magnetic bead-based DNA nano-sensors, a novel fluorescent CRISPR/Cas12a system is developed for miR-155 detection. This system utilizes carboxyl-functionalized poly 9,9-bis 3′-N, N-dimethylamino-2,7-fluorene-2,7-(9,9-dioctylfluorene) NPs (c-PFN NPs) as the fluorescence donor and Au-PDA-Au NPs as the fluorescent acceptor. The amino-labeled single-strand S2 is captured using c-PFN NPs to form S2-PFN. The miRNA155 can trigger CRISPR/Cas12a to cleave S3 in the quenching probe. The fluorescence signal increases because digested S3-Au-PDA-Au cannot hybridize with S2-PFN [157].

#### 2.5.4. Mechanical

These beads, often at the nanoscale or microscale, serve as a platform for the immobilization of biological molecules such as antibodies, aptamers, or DNA probes. The mechanical properties of these beads can affect the overall performance and sensitivity of the biosensor. Key considerations include diffusion, shear force, and precipitation.

(a) Diffusion

Diffusion is the movement of molecules from an area of higher concentration to one of lower concentration. In bead-based biosensors, diffusion is essential for the transport of target molecules to the bead surface for binding. Efficient diffusion ensures a rapid and uniform distribution of analytes, optimizing the chances of successful biomolecular interactions on the bead surface. A microfluidic platform is developed to combine magnetic-based single bead trapping with acoustic micro-mixing for simultaneous detection of multiple cancer biomarkers, in which the acoustic microstreaming induced rapid-flow patterns, systematically testing different driving frequencies to optimize the mixing effect and minimize diffusion length scales. This platform shows the potential for rapid POC diagnostics with a 20 min detection time and impressive sensitivity compared with established cutoff values [158]. Upon introducing the target to the electrochemical DNA-based biosensor platform, diffusion alterations in the lung cancer biomarker ENOX2 induce a conformational shift in the aptamers. Then, the distance between the redox reporter and the gold electrode is measured to determine the ENOX2 quantity [111]. Thus, beads with controlled porosity enhance mass transfer, improving the overall sensitivity of the biosensor.

(b) Shear Forces

Shear forces affect the binding kinetics and stability of biomolecular interactions on the bead surface. Understanding shear forces is essential for optimizing the conditions that balance the efficient binding and potential dissociation of biomolecules. In microfluidic systems, controlling shear forces is critical to preventing non-specific binding and maintaining the specificity of the biosensor. Vaidyanathan et al. introduces a device incorporating planar and three-dimensional microtip electrodes for the capture and detection of protein biomarkers through fluorescence [159]. The tunable nanoshearing mechanism significantly enhances the specificity and sensitivity for multiple protein biomarkers in the serum, offering a promising avenue for early cancer detection. Li et al. studied breast cancer biomarkers and discovered the application of antibody-conjugated microbeads in a microfluidic chip [50]. Increased flow rates and shear stress are found to improve reaction times and sensitivity, marking a significant step forward in liquid biopsy technology. The introduction of microbeads disrupts the liquid sample’s laminar flow, leading to increased mass-transfer efficiency. This modification significantly boosts the antibody’s attachment onto the specific proteins, resulting in an amplified fluorescence signal. Consequently, the detection’s sensitivity and efficiency are markedly improved. Through careful optimization of the experimental parameters, they achieve a remarkably low LOD of 0.1 ng/mL in CEA and CA15-3 detection. Green et al. established a cancer-cell sorting system by using magnetic NPs (MNPs) labeled with EpCAM. It uses a combination of shear stress and immuno-affinity capture to isolate phenotypically unique CTCs based on EpCAM expression levels [160]. Thus, proper shear forces contribute to consistent and reproducible biosensor performance.

(c) Precipitation

LAMP is a nucleic acid amplification technique that enables the rapid and efficient amplification of specific DNA sequences under isothermal conditions. After the LAMP amplification step, the amplified DNA can be captured onto the beads, either directly through hybridization with complementary probes or indirectly through precipitation methods. Then, it is subjected to precipitation techniques for further enrichment and detection on the surface of functionalized beads. In an innovative work by KR Sreejith et al., LAMP is creatively utilized within a core–shell bead framework to identify the heightened expression of tyrosine kinase AXL, an important marker for various cancer types [161]. Utilizing a thermal cycler alongside a fluorescent observation setup for the core–shell bead-centered LAMP process, they discovered that samples with an initial presence of 1 × 10^3^ copies are notably amplified within 20 min in the core–shell beads, reaching peak fluorescence at the 60 min mark. The core–shell beads’ spherical configuration may offer an uncomplicated approach to concentrating and discerning light, thereby possibly further streamlining the optical system’s design. Q. Lin et al. further highlighted LAMP’s adaptability for cancer-biomarker detection [162]. They introduced a dual-modality approach that integrates LAMP with magnetic bead isolation. This dual-modality method involves magnetic beads modified with an anti-methyl cytosine antibody for the rapid enrichment of methylated DNA, specifically targeting the Septin9 gene in colorectal cancer. The process detects methylated DNA within 30 min. The method successfully identifies methylated DNA from HCT 116 cells ranging within 2–0.02 ng/μL, with an LOD at 0.02 ± 0.002 ng/μL (RSD: 9.75%). Progress in LAMP technology has demonstrated its efficacy in improving cancer biomarker identification by using bead-based biosensors. This suggests effective paths for swift and precise diagnostic methods.

## 3. Challenges and Future Perspectives

Bead-based biosensors have made significant advancements in cancer-biomarker detection, but some challenges still need to be addressed for their widespread adoption and further development. This Section discusses the key challenges to provide insights into the future perspectives of bead-based biosensors.

### 3.1. Standardization and Validation

The standardization and validation of bead-based biosensors are crucial to ensuring their reliability, reproducibility, and comparability across different platforms and laboratories [163]. The harmonization of assay protocols, reference materials, and quality-control measures is essential to establish consistent performance and facilitate the translation of bead-based biosensors into clinical practice. Collaborative efforts among researchers, clinicians, and regulatory bodies are needed to establish standardized guidelines and validation frameworks for bead-based biosensors in cancer-biomarker detection [164].

### 3.2. Biomarker Selection

The selection of appropriate biomarkers is crucial to the development of bead-based biosensors. Identifying biomarkers that are specific, sensitive, and clinically relevant is essential for accurate cancer diagnosis, prognosis, and treatment monitoring. Furthermore, the discovery and validation of novel biomarkers that can provide comprehensive information about the disease state or therapeutic response are required. Collaborative efforts between researchers and clinicians are needed to identify and validate biomarkers that can effectively guide clinical decision making [165].

### 3.3. Integration with Complementary Technologies

The integration of bead-based biosensors with complementary technologies such as microfluidics, nanotechnology, and data analysis algorithms holds great promise for improving their performance and functionality [166]. Future research should focus on developing integrated systems that enable the sample processing, multiplexed analysis, and real-time monitoring of cancer biomarkers. Integration with emerging technologies such as artificial intelligence and machine learning can enhance analytical capabilities and enable predictive modeling for personalized cancer care [167].

### 3.4. Translation into Clinical Practice

The translation of bead-based biosensors from the research laboratory to clinical practice is a significant challenge. It requires overcoming regulatory hurdles, demonstrating clinical utility and cost effectiveness, and addressing logistical considerations [168]. Close collaboration among researchers, clinicians, regulatory agencies, and industry partners is essential to facilitate the clinical validation, regulatory approval, and commercialization of bead-based biosensors. Clinical studies and large-scale trials are necessary to establish the clinical performance, patient outcomes, and economic value of these biosensors [169].

### 3.5. Accessibility and Affordability

Ensuring the accessibility and affordability of bead-based biosensors is crucial for their global impact, especially in resource-limited settings. Cost-effective fabrication techniques, scalable manufacturing processes, and affordable readout systems are needed to make these biosensors accessible to wide-ranging healthcare settings [16]. Additionally, healthcare professionals should be trained in the use and interpretation of bead-based biosensors to maximize their impact in diverse healthcare settings.

Bead-based biosensors hold great potential for cancer-biomarker detection. However, several challenges need to be addressed for their successful integration into clinical practice. Standardization, biomarker selection, integration with complementary technologies, translation into clinical settings, and accessibility are key areas that require attention [170]. Overcoming these challenges can pave the way for the widespread adoption of bead-based biosensors and their integration into routine cancer diagnostics, ultimately improving patient outcomes and personalized cancer care.

## 4. Conclusions

We review bead-based biosensors designed to identify different biomarkers crucial to advancing cancer diagnostics. This study investigates the multifaceted functions of different materials used in bead-based biosensors and their respective impacts on the overall performance of the biosensor system. Furthermore, we conduct a comprehensive analysis of the signal-amplification strategies used in these biosensors, aiming to elucidate their mechanisms and evaluate their effectiveness in enhancing detection sensitivity and accuracy.

The primary objective of multianalyte biosensors in cancer diagnostics is to enable the sensitive, specific, and cost-effective detection of biomarkers for clinical applications. Liquid biopsy offers an attractive approach to identifying various biomarkers, including circulating tumor cells, cfDNA, and extracellular vesicles. However, the current state of these biosensors primarily involves hypothesis testing or validation in the laboratory phase, with limited practical application as a cancer diagnostic tool. Challenges related to assay requirements and technical variability in pre-analytical steps hinder their seamless transition into clinical practice. Many biosensors still rely on multiple manual steps for sensing various analytes; some even omit the essential sample preparation process.

Despite these obstacles, the detection of multiple cancer biomarkers holds tremendous potential for enhancing clinical diagnostics, especially in early cancer biomarker identification, personalized therapy, and therapy monitoring. Accordingly, future research should prioritize three key areas: (i) identifying specific combinations of biomarkers capable of determining the origin, status, and progression of cancer, (ii) developing sample-to-answer biosensors that enable the simultaneous detection of diverse cancer biomarkers, and (iii) translating this information into a clinically relevant format.

One promising avenue for advancing multianalyte biosensors is the integration of bead-based technology. Bead-based biosensors offer the unique advantages of enhanced sensitivity and specificity in simultaneously detecting multiple analytes. Optical, electrochemical, and magnetic bead-based biosensors each have unique advantages in sensitivity, multiplexing capabilities, and compatibility with different readout systems. The choice of the appropriate bead-based biosensor depends on specific application requirements, such as desired sensitivity, multiplexing capability, and availability of instrumentation. By leveraging this technology, researchers can potentially address the challenges associated with manual steps and sample preparation, paving the way for more streamlined and efficient biosensor applications in cancer diagnostics. Bridging the gap between theoretical advancements and practical implementation is crucial so that these innovative biosensors into can be routinely used in clinical settings.

Numerous studies have emphasized the efficacy of detecting multiple analytes, particularly in the context of cancer. A significant example is the concurrent identification of cancer protein biomarkers alongside circulating tumor DNA/RNA or exosomes, which demonstrates substantial advantages in cancer screening and early detection. This approach is valuable for ensuring accurate diagnosis, predicting patient prognosis, assessing therapy response, and monitoring cancer progression. The comprehensive insights gained through multianalyte detection can empower healthcare professionals to make targeted and well-informed decisions regarding therapy.

Despite the promising outcomes for bead-based biosensors, the majority of these biosensors notably remain in the research or integration phase. Currently, no product for routine clinical use is commercially available. This absence underscores the need for the further development and translation of these innovative technologies from the research setting to practical applications in clinical diagnostics.

Regardless of existing challenges in standardization, biomarker selection, and clinical translation, continued research and development in this field holds great promise. With further advancements and collaborations, bead-based biosensors have the potential to revolutionize cancer diagnostics, enabling early detection and personalized treatment.

## Figures and Tables

**Table 1:** Common cancer biomarkers detectable by bead-based biosensors.

Cancer Type	Biomarkers	References
Breast Cancer	HER2, ER, BRCA1, BRCA2, CA15-3	[8,9,10]
Lung Cancer	CYFRA21-1, LncRNA MEG3, EGFR, PD-L1, KRAS mutation	[11,12,13,14,15]
Colorectal Cancer	CA19-9, EGFR, CEA	[16,17,18]
Prostate Cancer	PSA, LncRNA PCA3, PAP	[19,20,21]
Gastric Cancer	HER2, PD-L1, CA72-4, CEA	[22]
Hepatic Cancer	AFP, AFP-L3, DCP	[23]
Pancreatic Cancer	CA19-9, CEA, KRAS, TP53	[24]
Ovarian cancer	CA-125, BRCA1, BRCA2, CA 19-9, AFP	[25]
Leukemia	BCR-ABL	[26]

HER2: Human Epidermal Growth Factor Receptor 2, ER: Estrogen Receptor, BRCA1: Breast Cancer Gene 1, BRCA2: Breast Cancer Gene 2, CA15-3: Cancer Antigen 15-3, CYFRA21-1: Cytokeratin 19 Fragment, LncRNA MEG3: Long Non-Coding RNA Maternally Expressed Gene 3, EGFR: Epidermal Growth Factor Receptor, PD-L1: Programmed Death-Ligand 1, KRAS mutation: Kirsten Rat Sarcoma Viral Oncogene Mutation, CA19-9: Carbohydrate Antigen 19-9, CEA: Carcinoembryonic Antigen, PSA: Prostate-Specific Antigen, LncRNA PCA3: Long Non-Coding RNA Prostate Cancer Gene 3, PAP: Prostatic Acid Phosphatase, CA72-4: Cancer Antigen 72-4, AFP: Alpha-Fetoprotein, AFP-L3: Alpha-Fetoprotein-L3 Fraction, DCP: Des-γ-Carboxy Prothrombin, TP53: Tumor Protein p53, CA-125: Cancer Antigen 125, BCR-ABL: Breakpoint Cluster Region-Abelson Murine Leukemia Viral Oncogene Homolog 1.

**Table 2:** Advantages and disadvantages of bead-based biosensors.

Pros	Cons
✓ High Surface to Volume Ratio ✓ High Throughput ✓ Fast Response ✓ Reduced Matrix Effects ✓ Enhanced Sensitivity and Dynamic Range ✓ Multiplexed Detection ✓ Minimal Sample Volume ✓ Miniaturization and Portability	Reproducibility and Standardization Cross-Reactivity Instrumentation Complexity Clinical Translation

**Table 3:** Signal amplification of bead-based biosensors for cancer biomarker detection.

Type	Biomarker	Detection Limit	Linear Range	Reference
Enzymatic amplification	CEA	6.12 pg/mL	0.02–50 ng/mL	[18]
	CEA	4.0 pg/mL	0.01–6.0 ng/mL	[55]
Nucleic acid amplification	AFB1	0.13 pg/mL	0.5–40 pg/mL	[44]
	miRNA16	8.81 fM	10 fM–100 pM	[45]
	miRNA21	3.85 fM	10 fM–1 nM	
Ultra-sensitive probes	CEA	30.5 fg/mL	100 fg/mL–50 ng/mL	[36]
	DNA	0.5 pM	10 pM–50 nM	[37]
	EN2	0.34 nM	3.12–50 nM	[38]
Enrichment by concentration	CEA/CA15-3	0.1 ng/mL	0.2–30 ng/mL	[50]
	CEA	3.0 pg/mL	0.01−20 ng/mL	[51]
Others	PSA	10 fg/mL	100 fg/mL–10 ng/mL	[39]
	HER2	0.37 pg/mL	0.75–250 pg/mL	[40]

CEA: carcinoembryonic antigen; AFB1: aptamer of aflatoxin B1; miRNA: microRNA; EN2: Engrailed-2; CA15-3PSA: cancer antigen 15-3, prostate specific antigen; HER2: human epidermal growth factor receptor 2.

**Table 4:** Optical bead-based biosensors for cancer biomarkers diagnosis.

Sensing Mechanism	Target Biomarker	Detection Elements	Signal Elements	Detection Limit	Reference
Fluorescence	HER2	HER2 Ab	FITC	0.004 ng/mL	[113]
	CA125	CA125 Ab	Cy5	0.005 U/mL	
	CA125	CA125 aptamer	Near-infrared photoluminescence	0.07 ng/mL	[114]
	CA125	CA125 aptamer & CA125 Ab	Fluorescence resonance energy transfer	400 cells/mL	[115]
	MUC1	DNA probe & MUC1 Ab	CdTe QDs & Fluorescence-AMCA	-	[116]
	MUC1	biotin-labeled aptamer	Fluorescence of CdZnTeS QDs	0.13 ng/mL	[117]
SPR	CA125	CA125 Ab	11-mercaptoundecanoic acid	0.1 U/mL	[120]
	CA125	CA125 Ab	Gold nanorod	0.4 U/mL	[121]
	HE4	HE4 Ab	Nanochip	4 pM	[122]
Raman spectroscopy	MUC1	MUC1 aptamer	4-mercaptopyridine	-	[124]
	BRAF & PIK3CA	Gene probes	Fluorescence label	10^−11^ M	[125]
	miR-223	DNA probes	Raman reporters	10^−17^ M	[126]
	AFP	AFP Ab		10^−12^ M	
	CD47 & CA9	CD47 Ab & CA9 Ab	Raman dyes	-	[108]
	miR-10b	DNA probes	DTNB	10^−18^ M	[127]

**Table 5:** Electrochemical biosensors for detecting cancer biomarkers.

Cancer Type	Electrode	Target Biomarker	Detection Limit	Linear Range	Reference
Breast cancer	Dye labeled DNA probe	CA15-3	0.0039 U/mL	0.01–1 U/mL	[10]
	Oligonucleotides modified probe	BRCA1	1.72 fM	50.0 fM–1.0 nM	[132]
	Ferrocenecored poly (amidoamine) dendrimers	BRCA1	0.38 nM	1.3–20 nM	[133]
	Apt-EGFR-Ab/MB	EGFR	50 pg/mL	1–40 ng/mL	[136]
	HER2 probe & CD24c DNA modified probe	HER2	0.16 nM	0.37–10 nM	[137]
Lung cancer	ssDNA modified probe	CYFRA21-1	1.0 × 10^−14^ M	10 fM–100 nM	[11]
	ssDNA modified probe	EGFR	120 nM	0.1 μM–3 μM	[13]
	Primer probes	MEG3	0.25 fM	1 fM–100 pM	[12]
	GCE/G2Fc/Ab	IgG	2.0 ng/mL	5.0–50 ng/mL	[138]
	graphene, chitosan and glutaraldehyde	CYFRA21-1	43 pg/mL	0.1 to 150 ng/mL	[139]
Prostate cancer	polypyrrole/Au/Ab	PSA	0.3 fg/mL	10 fg/mL–10 ng/mL	[19]
	FTO/SWCNTs/Au/probe	miR-21	0.01 fM L^−1^	0.01 fM–1 μM	[140]
Ovarian cancer	Polyaniline	CA125	0.923 ng/μL	0.92 pg/μL–15.20 ng/μL	[25]
	Ag NPs-GQDs	Mucin-16	0.01 U/mL	0.01–400 U/mL	[142]
	Au-Ag nanoparticles	CA125	5.9 IU/mL	1–150 IU/mL	[143]
Universal	Fe_2_N@rGOS/probe	4-NQO	9.24 nM	0.05–574.2 μM	[144]
	p53-Ab2-tGO-AuNPs	p53	4 fg/mL	20–1000 fg/mL	[145]
	glassy carbon	MUC1	2.7 nM	5–115 nM	[148]
	Ab1/rGO-AuNPs/GCE	CEA	5.3 pg/mL	50–650 pg/mL	[149]
	ITO-PET/EDC-NHS/Ab	SOX2	7 fg/mL	25 fg/mL–2 pg/mL	[150]
	Au NPs/TB–GO/probe	MDR1	2.95 × 10^−12^ M	0.01–1.0 nM	[151]
	SOI/SiNW/PhNO_2_/Ab	8-OHdG	1 ng/mL	1–40 ng/mL	[152]

BRCA1: Breast cancer type 1 susceptibility protein; EGFR: Epidermal growth factor receptor; HER2: Human epidermal growth factor receptor 2; ssDNA: single strand DNA; CYFRA21-1: Cytokeratin 19 fragment 21-1; PSA: Prostate-specific antigen; miR-21: micro RNA 21; FTO: Fluorine-doped tin oxide; SWCNTs: single-wall carbon nanotubes; CA125: Cancer Antigen 125; 4-NQO: 4-nitroquinoline 1-oxide; MUC1: Mucin 1; NPs: Nanoparticles; CEA: Carcinoembryonic Antigen; SOX2: SRY-Box transcription factor 2; MDR1: Multidrug Resistance 1; SOI: silicon-on-insulator; 8-OHdG: 8-hydroxydeoxyguanosine.

## Data Availability

The original contributions presented in the study are included in the article, further inquiries can be directed to the corresponding author/s.
